# SARS-CoV-2 Multi-Antigen Serology Assay

**DOI:** 10.3390/mps4040072

**Published:** 2021-10-09

**Authors:** Ramin Mazhari, Shazia Ruybal-Pesántez, Fiona Angrisano, Nicholas Kiernan-Walker, Stephanie Hyslop, Rhea J. Longley, Caitlin Bourke, Catherine Chen, Deborah A. Williamson, Leanne J. Robinson, Ivo Mueller, Emily M. Eriksson

**Affiliations:** 1 Walter and Eliza Hall Institute of Medical Research, Melbourne, VIC 3052, Australia; mazhari@wehi.edu.au (R.M.); ruybal.s@wehi.edu.au (S.R.-P.); walker.n@wehi.edu.au (N.K.-W.); rosiehyslop@live.com.au (S.H.); longley.r@wehi.edu.au (R.J.L.); bourke.c@wehi.edu.au (C.B.); chen.ca@wehi.edu.au (C.C.); leanne.robinson@burnet.edu.au (L.J.R.); mueller@wehi.edu.au (I.M.); 2 Department of Medical Biology, The University of Melbourne, Melbourne, VIC 3052, Australia; 3 Vector-Borne Diseases and Tropical Public Health, Burnet Institute, Melbourne, VIC 3004, Australia; fiona.angrisano@burnet.edu.au; 4 Royal Melbourne Hospital, Melbourne, VIC 3052, Australia; deborah.williamson@unimelb.edu.au; 5 Public Health Laboratory, Peter Doherty Institute for Infection and Immunity, Melbourne, VIC 3052, Australia

**Keywords:** SARS-CoV-2, COVID-19, Luminex, serology

## Abstract

Serology tests are extremely useful for assessing whether a person has been infected with a pathogen. Since the onset of the COVID-19 pandemic, measurement of anti-SARS-CoV-2-specific antibodies has been considered an essential tool in identifying seropositive individuals and thereby understanding the extent of transmission in communities. The Luminex system is a bead-based technology that has the capacity to assess multiple antigens simultaneously using very low sample volumes and is ideal for high-throughput studies. We have adapted this technology to develop a COVID-19 multi-antigen serological assay. This protocol described here carefully outlines recommended steps to optimize and establish this method for COVID-19-specific antibody measurement in plasma and in saliva. However, the protocol can easily be customized and thus the assay is broadly applicable to measure antibodies to other pathogens.

## 1. Introduction

The pathogen causing coronavirus disease 2019 (COVID-19) has been identified as a novel zoonotic coronavirus termed the severe acute respiratory syndrome coronavirus (SARS-CoV-2). Disease severity in individuals varies dramatically and ranges from asymptomatic disease to severe disease requiring hospitalization, oxygen supplementation, and intubation, and to death. IgG and IgM anti-SARS-CoV-2 antibodies are generated in most infected individuals, though mild and asymptomatic infections result in relatively low antibody levels [1]. Kinetic studies to date suggest that both IgM and IgG anti-SARS-CoV-2 antibodies are detectable approximately 7–10 days after the onset of clinical symptoms, although the longevity of IgM is shorter than IgG [2]. Longitudinal studies have demonstrated the presence of antibodies and the neutralizing effects of these antibodies after 8–10 months in people who have recovered from both mild and severe COVID-19 disease [3,4]. Despite a high seroprevalence in Manaus, Brazil after a first infection wave, a resurgence of cases ensued 7–8 months later with very high attack rates [5], highlighting the challenges posed by waning immunity and newly emerging viral variants. There have also been indications that pre-existing antibodies to seasonal coronaviruses may confer some level of protective immunity to severe COVID-19 disease [6,7].

IgA is also detected in SARS-CoV-2-infected individuals, both in plasma and in saliva, and has been suggested to provide early neutralization of the virus. Similar to IgM, IgA antibodies have shorter longevity in plasma than IgG antibodies, although SARS-CoV-2-specific IgA antibodies in saliva have been described to persist for longer than in plasma [8]. This suggests that serological assessments require measurement of complex antibody signatures of multiple isotypes to establish not only that infection has occurred, but also to provide information on the timing of previous exposure to SARS-CoV-2 and presence of co-factors that may be beneficial to allow estimation of both individual immune status and population immunity levels.

The Luminex xMAP technology is based on commercially available COOH non-magnetic or magnetic beads that are read on a compatible Luminex instrument [9]. Beads are internally colour coded, which results in spectrally distinct profiles enabling identification of separate bead sets. As each antigen is covalently coupled to a specific bead set, antibody levels to multiple antigens can be assessed simultaneously in single plasma, serum, or saliva samples. Luminex-based serology assays have been effectively used to measure antigen-specific antibodies to several pathogens, including malaria, HIV and now also respiratory viruses [10,11,12,13,14,15,16,17,18,19]. The advantage of this assay on a MAGPIX system is that a small sample volume of as little as 1 µL can be used for the detection of up to 50 antigens using magnetic beads (and up to 500 antigens on the Flexmap). The ability to measure responses to multiple antigens could be particularly useful in distinguishing between vaccine-induced responses that rely on antibodies to Spike only and antibody responses to natural infection which will also induce antibody responses to non-Spike proteins. Here, we report on the adaptation and development of the Luminex-based assay to measure IgG, IgM and IgA antibody levels to a panel of respiratory viruses consisting of SARS-CoV-2, seasonal coronaviruses caused by the following four human coronaviruses (HKU1, 229E, OC43 and NL63) and influenza virus strains A and B. In this protocol, we describe how to achieve the best signal-to-noise readout and optimal assay performance to detect various antibody levels to the selected viral antigens. We also demonstrate that this assay is easily adaptable to measuring antigen-specific antibodies in other bodily fluids such as saliva. The detailed description of the assay not only outlines important steps and considerations needed to successfully adapt the same assay for other laboratories, but also provides a protocol that can easily be utilized to develop customized panels of antigens from other pathogens of interests.

## 2. Experimental Design

**Figure 1:**
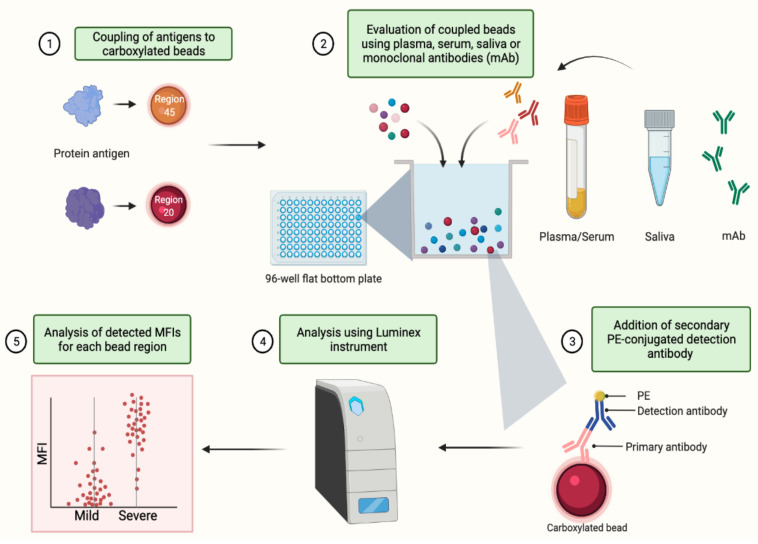
Schematic of serological assay workflow. Created with BioRender.com (accessed on 24 August 2021).

This protocol outlines steps required to optimize and set up a SARS-CoV-2 multi-antigen serological assay using magnetic carboxylated beads. The main experimental stages include coupling of SARS-CoV-2 protein antigens to the magnetic carboxylated beads (Bio-Rad, Hercules, CA, USA; time required approximately 3 h or overnight) and evaluation of the coupled beads using either a dilution series of an antibody positive plasma or saliva pool or using antigen-specific monoclonal or polyclonal antibodies (time required approximately 3–4 h/plate depending on antibody isotype being measured and the compatible Luminex instrument used) summarized in Figure 1.

### 2.1. Materials

- Phosphate-buffered saline (DPBS, pH 7.0–7.3 Gibco, Cat # 14190-144),
- Monobasic sodium phosphate buffer pH 6.0 (Sigma, St. Louis, MO, USA, Cat # S0751-500G),
- Sulfo-NHS (hydroxysulfosuccinimide sodium salt 98%, Sigma, St. Louis, MO, USA, Cat # 56485-250MG),
- EDC (*N*-Ethyl-*N*′-carbodiimide hydrochloride, Sigma, St. Louis, MO, USA, Cat # 03449-5G),
- Na-azide (Sodium azide, Sigma, St. Louis, MO, USA, Cat # S2002-25G),
- Tween-20 (Sigma, St. Louis, MO, USA Cat # P1379),
- MilliQ-water (MQ-H_2_O),
- PBT buffer (1× PBS, 1% BSA, 0.05% Tween-20; sterile filter the PBS-BSA prior to adding Tween-20),
- Bovine serum albumin (BSA, Sigma, St. Louis, MO, USA Cat # A7906-100G),
- Carboxylated microspheres (Bio-Plex Pro Magnetic COOH Beads, Bio-Rad, Hercules, CA, USA, 1 mL (1.25 × 10^7^ bead/mL), Cat # MC100X01),
- R-PE AffiniPure F(ab’)_2_ Fragment, donkey anti-human IgG (Jackson Immuno Research, West Grove, PA, USA, Cat # 709-116-098),
- R-PE AffiniPure F(ab’)_2_ Fragment, donkey anti-human IgM (Jackson Immuno Research, West Grove, PA, USA, Cat # 709-116-073),
- Goat F(ab’)_2_ anti-human IgA-PE (Southern Biotech, Birmingham, AL, USA, Cat # 2052-09),
- Antigens (Table 1),
- Positive control (plasma or saliva from COVID-19 diagnosed convalescent individuals),
- Negative control (naïve control plasma or saliva collected from individuals before the start of the pandemic or SARS-CoV-2 negative),
- 96-well round-bottom plate, clear (Corning, Corning, NY, USA, Cat # 353077),
- 96-well flat-bottom plate, black (Greiner Bio-One, Kremsmunster, Austria, Cat # 655090),
- Microcentrifuge tubes (Eppendorf, Safe-Lock tubes, Cat # 0030 120.191),
- Falcon tubes (15 mL Polystyrene conical tube, Corning, Corning, NY, USA, Cat # 352095),
- Magpix Calibration Kit (Luminex, Austin, TX, USA, Cat # MPX-CAL-K25),
- Magpix Verification Kit (Luminex, Austin, TX, USA, Cat # MPX-PVER-K25), and
- Magpix Drive Fluid (Luminex, Austin, TX, USA, Cat # MPX-DF-4PK).

### 2.2. Equipment

- Magnetic plate washer (BioTek Instruments, Winooski, VT, USA, BioTek ELx50),
- Bio-Rad Sure Beads magnetic rack (Bio-Rad, Hercules, CA, USA, Cat # 1614916),
- Ratek Platform shaker (Ratek Instruments, Boronia, Australia, MPS1 Microtiter/PCR Plate Shaker),
- Vortex Sonicator (Branson 2200),
- BioSan Vortex V-1 plus,
- Table centrifuge (Eppendorf Centrifuge 5424), and
- MAGPIX^®^ Multiplexing System (Millipore, Burlington, MA, USA, MAGPIX^®^ System, Xponent software V.4.2).

## 3. Procedure

### 3.1. Optimization of Coupling SARS-CoV-2 and Control Proteins to Magnetic Beads

SARS-CoV-2 and control recombinant protein antigens included in this study were either obtained commercially or kindly provided by collaborators. See Table 1 for a complete list of proteins, the protein expression system used (Human embryonic kidney cells 293-HEK 293, *E. coli* and Baculovirus-insect cells), supplier and relevant protein description. For the coupling of SARS-CoV-2 and control proteins, magnetic carboxylated beads were sourced from Bio-Rad (1 region/antigen, i.e., 14 different regions to be able to multiplex 14 different antigens in one assay) and stored at 2–4 °C.

The optimal protein amount to couple to magnetic beads was first determined. All proteins were initially coupled to 6.25 × 10^5^ pre-activated microspheres (Table 2) at various protein concentrations (trial coupling). The optimum antigen concentration was then determined by observing what concentration of protein resulted in a log-linear standard curve using the convalescence plasma or saliva pool. It is important to acquire a log-linear standard curve to achieve an optimal dynamic range to be able to measure a wide range of different concentrations whilst measuring all samples at the same dilution. Once determined, 2.5 × 10^6^ pre-activated microspheres were used to prepare a larger quantity of coupled antigens (bulk coupling) that can subsequently be used to screen plasma or saliva from approximately 3000 individuals with unknown COVID-19 status.

#### 3.1.1. Microsphere Activation

Minimize exposure of the magnetic beads to light by covering the beads in all steps as much as possible.

- Sonicate magnetic microsphere stocks for 15 s followed by 10 s vortexing, before transferring 50 µL beads in the trial coupling phase (6.25 × 10^5^ beads) and 200 µL beads for the bulk coupling phase (2.5 × 10^6^ beads) to a microcentrifuge tube.
- Place the tubes in a magnetic separator rack and allow separation to occur for 30–60 s.
- While tubes still positioned in the rack, remove the supernatant, taking care not to disturb the beads.
- Remove the tubes from the rack and resuspend beads in MQ-H_2_O (100 µL for trial coupling and 200 µL for bulk coupling) by vortexing for 20 s.
- Place tube back into magnetic separator rack for 30–60 s and while positioned in the rack remove supernatant, taking care not to disturb the beads.
- Remove the tubes from the rack and resuspend in 100 mM monobasic sodium phosphate buffer, pH 6.2 (80 µL for trial coupling and 160 µL for bulk coupling) and vortex for 20 s.
- Add sulfo-NHS (50 mg/mL in MQ-H_2_O), mix by gentle vortex for 10 s and add EDC (50 mg/mL in MQ-H_2_O), mix again by gentle vortex for 10 s (10 µL each for trial and 20 µL for bulk coupling) and incubate while rotating in the dark for 20 min, to activate the beads. 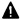 **CRITICAL STEP** Sulfo-NHS and EDC should be made fresh on the day of use.
- After the incubation, place the tubes into a magnetic separator rack for 30–60 s and while positioned in the rack, remove supernatant, taking care not to disturb the beads.
- Remove the tubes from the rack and resuspend in PBS, pH 7.0–7.3 (250 µL for trial and 500 µL for bulk coupling), vortex for 20 s and place in magnetic separator for 30–60 s and remove the supernatant.
- Remove tubes from magnetic separator and continue the washing with PBS one more time (for a total of 2 washes).
- Resuspend the activated and washed beads in PBS (250 µL volume for trial couplings and 500 µL volume for bulk coupling), vortex for 20 s and put aside for the protein coupling step.

#### 3.1.2. Protein Coupling to Microspheres

- Add the appropriate volume of each protein, which corresponds to the protein concentration being tested, to the activated beads and incubate while rotating in the dark for 2 h at room temperature. 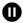 **PAUSE STEP** Beads can also be left over night at 4 °C, on a rotator.
- Place tubes again into a magnetic separator rack and allow separation for 30–60 s and remove supernatant without disturbing the beads.
- Resuspend the beads in 500 µL PBS-TBN (PBS, pH 7.0–7.3 plus 0.02% Tween-20, 0.1% BSA and 0.05% Na-azide), vortex for 20 s and repeat the washing two more times (three washes in total).
- After the final removal of supernatant, resuspend the beads in PBS-TBN (125 µL for trial couplings and 500 µL for bulk couplings), vortex for 10 s. **Note**: Bead loss is minimal when using magnetic beads and the magnetic separator racks and thus counting of beads after coupling are not required. However, a loss in bead numbers could occur due to the various centrifugation steps, when using non-magnetic beads. If non-magnetic beads are used, counting of the bead numbers under the microscope after final resuspension is recommended to achieve consistent final bead-concentration. 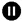 **PAUSE STEP** Store the coupled beads at 4 °C in the dark.

### 3.2. Evaluation of Capacity to Measure Antigen-Specific IgG, IgM and IgA Antibody Responses

The reactivity of the coupled beads is evaluated by a multiplex antibody assay, using a dilution series of an antibody positive plasma or saliva pool to obtain a log-linear standard curve with a relative high mean fluorescence intensity (MFI) of preferably 10,000 or higher. The plasma and the saliva pool used in this study consisted of convalescent plasma or saliva from 10 SARS-CoV-2 qPCR-positive individuals, collected approximately 30 days after onset of symptoms (plasma) or within the three first months after diagnosis (saliva). The naïve control plasma (*n* = 10) was collected from healthy individuals in June 2019 prior to the pandemic samples. Depending on linearity and the relative MFI of each coupled protein, the protein amount used for the trial couplings needs to be adjusted. Previous qualitative assessments have confirmed the stability of protein-coupled beads by comparison of the MFI of the standard curve over a nine-month period [9], though this may vary depending on the protein.

To assess the reactivity of antibodies to the selected proteins, antigen-specific IgG, IgM or IgA was detected by incubating 500 beads of each antigen per well with plasma at a 1:200 final dilution or saliva at a 1:10 final dilution. **Note:** The saliva sample dilution was established based on optimizations performed previously to achieve a log-linear standard curve with saturated beads (Table 2) and to allow the dilution factor to be kept constant across samples. Sample dilutions may vary for different panels.

**Figure 2:**
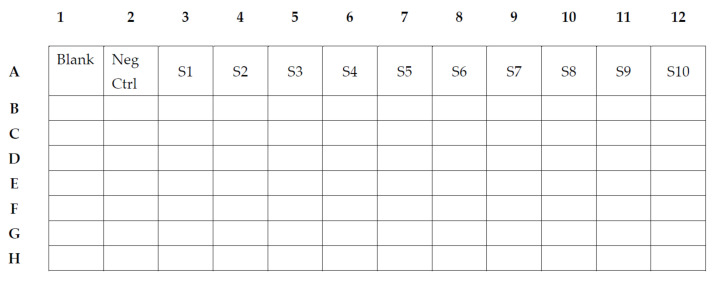
An example of plate layout containing a blank and negative control as well as a standard curve (S1–S10). Remaining wells are used for individual samples.

- Saliva is pre-processed by centrifugation in a table centrifuge at 10,000× *g* for 15 min to remove cell pellets and debris. The saliva sample is then aliquoted before freezing at −80 °C until use.
- Samples and standard curves are pre-diluted in a 96-well round-bottom plate (dilution plate).
- In the 96-well round-bottom dilution plate, we use one well of blank, consisting of 100 µL PBT buffer (Figure 1).
- One well of negative control, consisting of 99 µL PBT and 1 µL of the negative plasma or 90 µL PBT and 10 µL of the negative saliva (Figure 2).
- In wells A3–A12, we add 75 µL PBT to each well, except in well A3, in which a total of 147 µL PBT and 3 µL of the positive plasma pool or 135 µL PBT and 15 µL of the positive saliva pool will be added to achieve a 1:50 dilution or 1:10 dilution, respectively (S1). Two-fold serial dilution of the standard is achieved by transferring 75 µL after thorough mixing from A3 to A4, and consecutively from each well to the next thereafter until A12. This will provide a dilution series of the positive pool from 1:50 to 1:25,600 (Figure 2(S1–S10)) and 1:10 to 1:5120 for a saliva pool.
- Individual samples to be analyzed are pre-diluted in PBT (99 µL for plasma samples and 80 µL for saliva samples) in the remaining wells of the 96-well round-bottom dilution plate. Plasma (1 µL/well) or saliva (20 µL/well) is added to yield a 1:100 dilution and 1:5 dilution, respectively.
- Magnetic beads are prepared and added to a 96-well flat-bottom plate (assay plate). The magnetic bead mix is prepared by vortexing the individual coupled bead regions for 30 s each, before adding 0.1 µL/well of each (total amount is based on the number of wells used) to a total of 50 µL PBT/well in a master bead mix. A volume of 50 µL PBT, containing 0.1 µL of each of the coupled beads, are added to each well of the 96-well flat-bottom assay plate.
- After the addition of the bead mix to all wells, 50 µL of the plasma or saliva dilutions and control dilutions are added to each well on the black flat-bottom assay plate. This mix of coupled beads and plasma/saliva samples is incubated for 30 min (IgG and IgM) or 2 h (IgA) at room temperature on a shaker in the dark, followed by washing the plate 3× with 100 µL PBT, using a magnetic plate washer or a hand magnet.
- After the washings, add 100 µL of a 1:100 dilution of PE-conjugated Donkey F(ab’)_2_ anti-human IgG, IgM or IgA to the plate, mix well and incubate for 15 min at room temperature on a shaker in the dark.
- After a further washing of the plate, add 80 µL PBT to each well, mix well and keep on a shaker until plate is inserted and read on a compatible Luminex instrument.
- At least 15 beads of each region/antigen are then acquired and analyzed.

The results are expressed as the mean fluorescence intensity (MFI) of at least 15 beads for each antigen in a log-linear standard curve. The standard curve on each plate is used to convert each individual MFI value of the unknown samples to a relative antibody unit (RAU), which enables comparison between samples assessed in separate experiments and plates [9].

## 4. Expected Results

The basis of the assay relies on the attachment of proteins to the beads, after these have been activated as described before. As proteins vary widely in their structure, purity and the methodology in which they have been expressed, careful optimization for each antigen is required. The optimum antigen concentration is established when a log-linear standard curve is achieved, aiming for a relatively high MFI value for the 1:50 dilution or 1:10 dilution (preferably over 10,000), using the convalescence plasma or saliva pool. The range of protein concentrations tested, and the determined optimal concentration required is detailed in Table 2.

**Figure 3:**
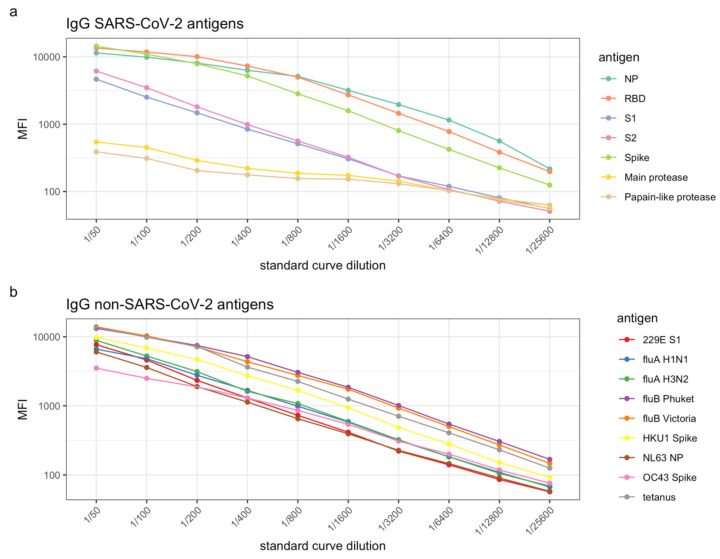
Standard curves for (**a**) SARS-CoV-2 antigens and (**b**) other antigens in human plasma, such as respiratory viruses and human seasonal coronaviruses. Levels of IgG antibodies are presented as log10-transformed median fluorescence intensity (MFI) values.

Optimal standard curves for SARS-CoV-2 antigens (NP, RBD, S1, S2, and Spike) and other antigens (influenza A (H1N1 and H3N2), influenza B (Phuket and Victoria), seasonal coronaviruses NL63 NP, OC43 Spike, 229E S1 and HKU1 Spike, as well as tetanus toxoid) are presented in Figure 3. The reactivity of antibodies to PLpro and the main protease of SARS-CoV-2 were also assessed (Figure 3a). However, the MFIs for the standard curves for these antigens were considerably lower than that of the other antigens. Given that IgG is the most abundant immunoglobulin in plasma, antigens which measure an MFI of <2000 produced with plasma from individuals known to have seroconverted and generated a robust antibody response are considered less or non-immunogenic. Antibodies specific for PLpro and main protease were concluded to not be present in convalescent plasma at substantial levels and therefore these antigens were excluded from the panel.

**Figure 4:**
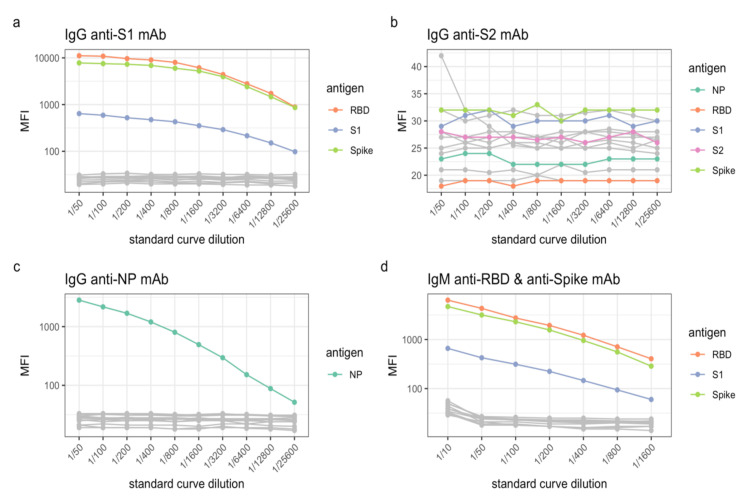
Reactivity of (**a**) anti-S1, (**b**) anti-S2, (**c**) anti-NP and (**d**) anti-RBD and anti-Spike human monoclonal antibodies to our 14-antigen panel. Levels of IgG or IgM antibodies are presented as log10-transformed median fluorescence intensity (MFI) values. Only the reactive and/or cross-reactive antigens are highlighted, all other antigens are in grey (including the seasonal coronaviruses, tetanus toxoid and influenza viruses).

We utilized a pool consisting of convalescent plasma from individuals with confirmed SARS-CoV-2 to establish the standard curves and a plasma pool consisting of plasma collected from individuals prior to the start of the COVID-19 pandemic in 2019 to determine background levels accounting for non-specific binding to antigen and beads. However, plasma may not always be readily available and human monoclonal antibodies can be used as an alternative approach to establish optimal standard curves. Here, we assessed the capacity of commercially available human monoclonal antibodies to recognize SARS-CoV-2 S1, S2 and NP antigens in our panel (Figure 4). The anti-S1 IgG antibody (Cat# MBS8574746, MyBiosource) bound to S1, but also recognized RBD which is part of the S1 domain, and Spike, which contains the S1 domain (Figure 4a). As expected, anti-NP IgG (Cat# MBS8574743, MyBiosource) bound the NP antigen in our panel (Figure 4c). In contrast, anti-S2 IgG (Cat# MBS8574747, MyBiosource) did not bind to the S2 protein antigen or any of the other SARS-CoV-2 (Figure 4b). In addition, we also assessed an IgM monoclonal antibody to Spike and RBD (Cat# srbd-mab5, InvivoGen).

The MFIs of the bound antibody for both antigens were comparable, but S1 was recognized to a lesser extent (Figure 4d). None of the monoclonal antibodies were cross-reactive to any of the seasonal coronavirus antigens. Furthermore, non-specific binding of the monoclonal antibodies to the influenza antigens was not observed (Figure 4a–d).

**Figure 5:**
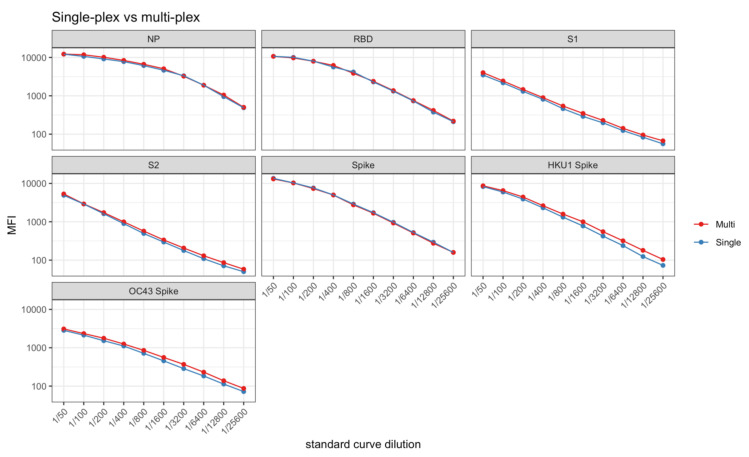
Comparison of standard curves in multiplex (red) and single-plex (blue). Levels of IgG antibodies in human plasma are presented as log10-transformed median fluorescence intensity (MFI) values.

Considering that S1, RBD and Spike as well as S2 are overlapping regions, multiplexing a panel that consists of these closely related antigens may result in competition and antibody levels to certain antigens may incorrectly appear lower than the actual levels. Similarly, cross-reactivity to corresponding antigens of related pathogens (e.g., OC43 Spike and SARS-CoV-2 Spike) may affect accurate measurement of these antigen-specific antibody levels. To address this in the current panel, we compared the standard curves for SARS-CoV-2 antigens (Spike, S1, S2, RBD and NP) and seasonal coronavirus antigens OC43 Spike and HKU1 Spike, when all the other antigens were included (multiplex) to when they were assessed separately (single-plex). There was no difference between the multiplex and the single-plex total IgG standard curves using human plasma for any of the antigens (Figure 5).

**Figure 6:**
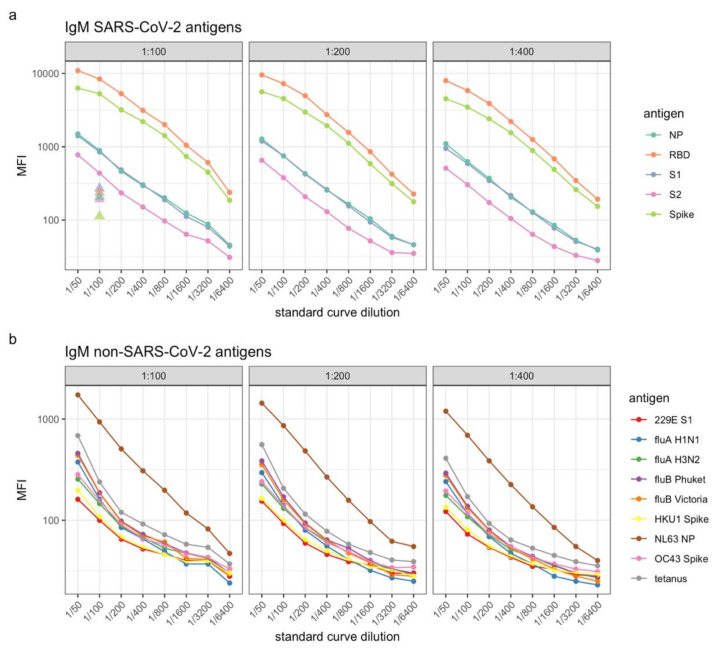
Standard curves for (**a**) SARS-CoV-2 antigens and (**b**) other antigens, such as respiratory viruses and human seasonal coronaviruses. Levels of IgM antibodies are presented as log10-transformed median fluorescence intensity (MFI) values. Solid triangles depict the negative values for each SARS-CoV-2 antigen, run at a 1:100 dilution.

IgM may be more relevant to assess in plasma collected at a relatively early time point after diagnosis. To test the adaptability of our panel to measure antigen-specific IgM levels, we used an anti-IgM detector antibody at various dilutions (1:100, 1:200 and 1:400; Figure 6). Standard curves for SARS-CoV-2 antigens NP and Spike indicated presence of comparable levels of IgM to IgG (Figure 3a). In contrast, MFIs for the remaining SARS-CoV-2 antigens and other respiratory pathogen antigens were low (Figure 6a,b).

**Figure 7:**
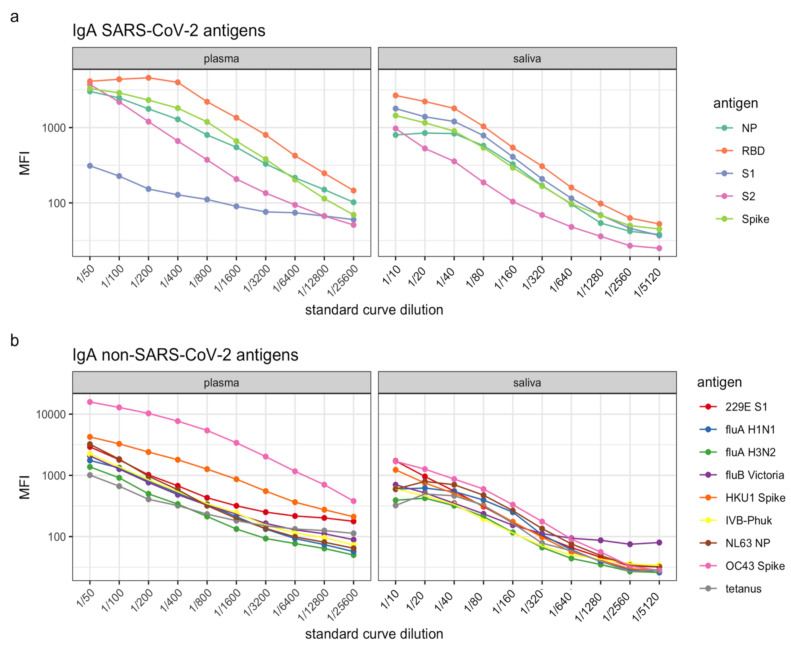
Standard curves for (**a**) SARS-CoV-2 antigens and (**b**) other antigens, such as respiratory viruses and human seasonal coronaviruses in plasma (left panels) and saliva (right panels). Levels of IgA antibodies are presented as log10-transformed median fluorescence intensity (MFI) values.

IgA is the predominant antibody isotype at mucosal surfaces such as the respiratory tract and may therefore be a better indicator of immunity to SARS-CoV-2. However, bronchoalveolar lavage fluid may not always be easily available. Saliva is a readily accessible, non-invasive alternative to respiratory fluid. Notably, SARS-CoV-2-specific IgA in plasma is also detectable, albeit reported to wane quickly [8]. We show here that using our assay, we can detect SARS-CoV-2 IgA antigen-specific antibodies in plasma as well as saliva (Figure 7a). Of note, IgA antibodies recognizing S1 is clearly detectable in saliva, but is less prominent in plasma, revealing differences in the level of S1-antigen-specific IgA in the different compartments. In contrast, beads that have been optimized for use with saliva and then are assayed under the same conditions with plasma may cause some antigens to reach saturation (Figure 7a, RBD). It is therefore recommended to optimize coupling conditions running both plasma and saliva in parallel and select conditions that achieve log-linear standard curves in both compartments. If only plasma or saliva are to be assayed, then the antigen coupling can be adjusted and optimized, respectively. Antigen-specific IgA antibodies to seasonal coronavirus antigens, tetanus toxoid and influenza A and B antigens are also observed in plasma and saliva, although the MFI readout from saliva is considerably lower than for plasma for these antigens (Figure 7b).

## 5. Discussion

This protocol describes an easily adaptable serological assay to measure antibody levels to multiple antigens simultaneously. The antigen panel we describe here covalently couples recombinant proteins to the magnetic beads, but slightly modified protocols allow for other antigens such as peptides, to also be coupled to the beads [20].

Glycosylation is one of the most common post-translational modifications of proteins and is important for the folding of proteins. Folding of proteins in turn determines exposure of epitopes to antibodies [21]. Production of recombinant proteins commonly uses a range of expression systems, including *E. coli*, HEK cells and Baculovirus system. The level of glycosylation depends in part on the internal machinery of the cells including glycosyltransferase and availability of nucleotide-sugars [21]. It is well established that the glycosylation capabilities of different species differ significantly, resulting in varying glycosylation between recombinant proteins expressed in mammalian, yeast and insect cells [22]. Thus, recombinant proteins derived from *E. coli* or HEK293 cells may differ substantially in their exposure of antigenic sites, and it is recommended that careful optimization of proteins is carried out. In this protocol, we describe assessment of antibodies to either *E. coli* or HEK293-expressed SARS-CoV-2 NP (Figure 2). Antibody levels to this antigen from either protein expression system were comparable. However, antibody levels to an antigen are not specific for a single epitope, and therefore epitopes recognized in the two proteins used in this study may still differ. This may be particularly important to consider in circumstances where known positive plasma is limited or unavailable and monoclonal antibodies are used to provide a standard. As monoclonal antibodies only recognize a single epitope, the combination of protein antigen and monoclonal antibody may be more sensitive to recombinant protein expression system and epitope availability. This was observed in our data, where a monoclonal antibody to the S1 domain was able to recognize RBD and Spike proteins but was less efficient in binding to the S1 protein, the monoclonal antibody should have the capacity to efficiently bind all three recombinant proteins. However, our observations highlight that there are distinct differences in epitope availability even though these recombinant proteins were all expressed in HEK293 cells.

Despite the requirement for careful assessment of protein and monoclonal antibody compatibility, using monoclonal antibodies instead of plasma pool from individuals to provide a standard curve has a definite advantage. Given that the concentrations of the monoclonal antibody used in the wells are known, it is a better quantitative estimation of the number of antigen-specific antibodies in the experimental plasma samples. In contrast, using a plasma pool from individuals of known disease status allows for a more rapid and potentially more cost-effective approach to optimize protein coupling to the beads, but is a non-quantitative method, which results in antibody levels reported as relative antibody units derived from the standard curve.

Single-plex assays, where an antigen is immobilized to a solid matrix such as a microtiter plate (ELISA), usually require a sample volume equal to or larger than what is necessary for multiplex assays. In addition, it is well appreciated that measurements of multiple factors/cytokines/antigen-specific antibodies are more biologically relevant. In this way, multiplex assays save valuable patient/participant serum or plasma, while providing a comprehensive analysis of the sample. However, in antigen panels that include multiple domains or regions of a single protein, the signal for any given antigen of that protein may be diminished. This may occur if antibodies preferentially bind particular epitopes of one antigen, which is represented in several domains included in the panel. In the current panel described here, this would represent S1, S2, RBD and Spike. In these cases, it is recommended to compare the signal obtained from analysis of the individually coupled beads with the plasma pool and/or monoclonal antibody (single-plex) with the signal obtained from when all the beads are analyzed simultaneously (multiplex). We found that the signals for the antigens included in the described panel here were comparable when analyzed as single- or multiplex.

Methods to measure SARS-CoV-2-specific antibodies in saliva are advantageous due to the ease of collecting the sample and have been used during the pandemic to support epidemiological surveillance or as alternative diagnostic tools [23,24,25]. Nevertheless, the current approaches are commonly restricted to ELISA-based methods, which only measure saliva antibodies to single antigens at a time. We show that the multi-antigen assay described here is easily adaptable to measuring antigen-specific antibodies in saliva samples, with the only difference being that the starting concentration of saliva samples is less diluted (1:10 dilution compared to 1:100 dilution, that is used for plasma samples). Measuring IgA in saliva in future studies, to multiple antigens, may represent an important source for additional insight into serological immunity to SARS-CoV-2 at mucosal sites.

## Figures and Tables

**Table 1:** SARS-CoV-2 and control proteins used.

Pathogen	Antigen Region	Expression System	Supplier	Catalogue Number	Description
**SARS-CoV-2**	**S1**	**HEK293**	**The Native Antigen Company, Oxford, UK**	**REC31806**	**SARS-CoV-2-Spike Glycoprotein (S1), recombinant antigen AA 1–674**
**SARS-CoV-2**	**S2**	**HEK293**	**The Native Antigen Company, Oxford, UK**	**REC31807**	**SARS-CoV-2-Spike Glycoprotein (S2) recombinant antigen AA 685-1211**
SARS-CoV-2	NP	*E. coli*	ProSpec Protein Specialists, Rehovot, Israel	sars-013	SARS-CoV-2 Nucleocapsid protein, full length AA 1-419
SARS-CoV-2	NP	*E. coli*	MP Biomedicals, Santa Ana, CA, USA	8720501	SARS-CoV-2 Nucleocapsid protein, full length AA 1-431
**SARS-CoV-2**	**NP**	**HEK293**	**AcroBiosystems, Newark, DE, USA**	**NUN-C5227**	**SARS-CoV-2 Nucleocapsid protein, full length AA 1-419**
**SARS-CoV-2**	**S-RBD**	**HEK293**	Wai-Hong Tham (WEHI) *2	-	**SARS-CoV-2-Receptor Binding Domain (RBD) of S1**
**SARS-CoV-2**	**Spike (Trimer)**	**HEK293**	Adam Wheatley (PDI) *3	-	**SARS-CoV-2 Full length Spike trimer**
SARS-CoV-2	Plpro *1	*E. coli*	David Komander (WEHI) *2	-	SARS-CoV-2-Papain-like protease
SARS-CoV-2	Main protease	*E. coli*	Peter Czabotar (WEHI) *2	-	SARS-CoV-2-Main protease
SARS	S1	HEK293	The Native Antigen Company, Oxford, UK	REC31809	SARS-CoV-1 S1 subunit
SARS	S1-RBD	HEK	Wai-Hong Tham (WEHI) *2	-	
MERS	S1	*E. coli*	ProSpec Protein Specialists, Rehovot, Israel	sars-002	Recombinant S1 subunit AA 56-295
**Seasonal Coronaviruses**					
HCoV-229E	NP	*E. coli*	The Native Antigen Company, Oxford, UK	REC31758	Recombinant NP, 359 AA
**HCoV-NL63**	**NP**	***E. coli***	**ProSpec Protein Specialists, Rehovot, Israel**	**sars-003**	**Recombinant c-terminal NP, contains 130 AA**
HCoV-NL63	S1	HEK293	Sino Biological, Beijing, China	40600-V08H	Recombinant S1 subunit, AA 13–756
**HCoV-229E**	**S1**	**HEK293**	**Sino Biological, Beijing, China**	**40601-V08H**	**Recombinant S1 subunit, AA 19-717**
HCoV-HKU1	S1	HEK293	Sino Biological, Beijing, China	40602-V08H	Recombinant S1 subunit, AA 13-756
**HCoV-HKU1**	**Spike**	**HEK293**	Adam Wheatley (PDI) *3	-	
**HCoV-OC43**	**S1 + S2 + ECD**	**Bac-Ins cells**	**Sino Biological, Beijing, China**	**40607-V08B**	**Recombinant Spike protein (S1 + S2 + ECD), AA 1-1304**
**Influenza Viruses**					
IVA Michigan H1N1-NA-45/2015	Neuraminidase	HEK293	Sino Biological, Beijing, China	40568-V07H	Influenza A Neuraminidase, AA 36-469
**IVA Switzerland H3N2-2013**	**Hemagglutinin**	**HEK293**	**Sino Biological, Beijing, China**	**40497-V08H1**	**Influenza A HA1, AA 1-345**
**IVB Victoria 02/1987**	**Hemagglutinin**	**HEK293**	**Sino Biological, Beijing, China**	**40163-V08H**	**Influenza B HA1, AA 1-362**
**IVB Phuket 3073/2013**	**Hemagglutinin**	**HEK293**	**Sino Biological, Beijing, China**	**40498-V08H1**	**Influenza B HA1, AA 1-361**
**IVA California H1N1-HA**	**Hemagglutinin**	**HEK293**	Adam Wheatley (PDI) *3	-	**Influenza A HA1**
**Tetanus Toxoid**			**Sigma-Aldrich, St. Louis, MO, USA**	**582231-25UG**	**Clostridium tetani (formaldehyde inactivation)**

*1—Papain-like Protease; *2—The Walter and Eliza Hall Institute of Medical Research, Melbourne, Australia; *3—The Peter Doherty Institute for Infection and Immunity, Melbourne, Australia. Text in bold indicate proteins included in the final panel.

**Table 2:** SARS-CoV-2 protein amounts used for coupling to magnetic beads.

Pathogen	Antigen Region	Protein Range Tested (µg)	Optimized Trial Protein Concentration (µg Ag/6.25 × 10^5^ Beads)	Protein Bulk Coupling (µg Ag/2.5 × 10^6^ Beads)	Saturated Protein Concentration-Bulk Coupling (µg Ag/2.5 × 10^6^ Beads)
**SARS-CoV-2**	**S1**	0.1–10	**5**	**10**	**40**
**SARS-CoV-2**	**S2**	0.1–10	**5**	**10**	**40**
**SARS-CoV-2**	**NP**	0.1–5	**1**	**2**	
SARS-CoV-2	NP	0.1–5			
**SARS-CoV-2**	**NP**	0.1–5	**0.5**	**1**	**10**
**SARS-CoV-2**	**S-RBD**	0.2–5	**2.5**	**5**	**20**
**SARS-CoV-2**	**Spike (Trimer)**	0.1–5	**2.5**	**5**	**20**
SARS-CoV-2	Plpro *1	1–5	-		
SARS-CoV-2	Main protease	1–5	-		
SARS	S1	0.5–5	-		
SARS	S1-RBD	0.5–5	-		
MERS	S1	0.5–5	-		
**Seasonal Coronaviruses**					
HCoV-229E	NP	0.2–5	-		
**HCoV-NL63**	**NP**	0.5–5	**5**	**10**	**40**
HCoV-NL63	S1	1–5	-		
**HCoV-229E**	**S1**	**0.2–5**	**1**	**2**	**12**
HCoV-HKU1	S1	0.5–5	-		
**HCoV-HKU1**	**Spike**	**0.2–5**	**1**	**2**	**12**
**HCoV-OC43**	**S1 + S2 + ECD**	**0.1–5**	**0.25**	**0.5**	**4**
**Influenza Viruses**					
IVA Michigan H1N1-NA-45/2015	Neuraminidase	0.5–5	-		
**IVA Switzerland H3N2-2013**	**Hemagglutinin**	0.2–5	**1**	**2**	
**IVB Victoria 02/1987**	**Hemagglutinin**	0.2–5	**1**	**2**	
**IVB Phuket 3073/2013**	**Hemagglutinin**	0.2–5	**1**	**2**	
**IVA California H1N1-HA**	**Hemagglutinin**	0.2–5	**0.4**	**0.8**	**6**
**Tetanus Toxoid**		0.5–5	**1**	**2**	**8**

Text in bold indicate proteins included in the final panel. Gray text and blank table cells indicate that coupling with protein concentrations listed did not yield an optimal standard curve.*1—Papain-like Protease
